# *In silico* analysis of the substitution mutations and evolutionary trends of the SARS-CoV-2 structural proteins in Asia

**DOI:** 10.22038/IJBMS.2022.66649.14620

**Published:** 2022-11

**Authors:** Mohammad Abavisani, Karim Rahimian, Mansoor Kodori, Reza Khayami, Mahsa Mollapour Sisakht, Mohammadamin Mahmanzar, Zahra Meshkat

**Affiliations:** 1 Student Research Committee, Mashhad University of Medical Sciences, Mashhad, Iran; 2 Department of Microbiology and Virology, Faculty of Medicine, Mashhad University of Medical Sciences, Mashhad, Iran; 3 Bioinformatics and Computational Omics Lab (BioCOOL), Department of Biophysics, Faculty of Biological Sciences, Tarbiat Modares University,; 4 Tehran, Iran; 5 Non communicable Diseases Research Center, Bam University of Medical sciences, Bam, Iran; 6 Department of Medical Genetics and Molecular Medicine, Faculty of Medicine, Mashhad University of Medical Sciences, Mashhad, Iran; 7 Department of Biochemistry, Erasmus University Medical Center, P.O. Box 2040, 3000 CA Rotterdam, The Netherlands; 8 Department of Bioinformatics, Kish International Campus University of Tehran, Kish, Iran

**Keywords:** Asia, COVID-19, Evolutionary analysis, Genome-wide mutations, Mutations, SARS-CoV-2

## Abstract

**Objective(s):**

To address a highly mutable pathogen, mutations must be evaluated. SARS-CoV-2 involves changing infectivity, mortality, and treatment and vaccination susceptibility resulting from mutations.

**Materials and Methods:**

We investigated the Asian and worldwide samples of amino-acid sequences (AASs) for envelope (E), membrane (M), nucleocapsid (N), and spike (S) proteins from the announcement of the new coronavirus 2019 (COVID-19) up to January 2022. Sequence alignment to the Wuhan-2019 virus permits tracking mutations in Asian and global samples. Furthermore, we explored the evolutionary tendencies of structural protein mutations and compared the results between Asia and the globe.

**Results:**

The mutation analyses indicated that 5.81%, 70.63%, 26.59%, and 3.36% of Asian S, E, M, and N samples did not display any mutation. Additionally, the most relative mutations among the S, E, M, and N AASs occurred in the regions of 508 to 635 AA, 7 to 14 AA, 66 to 88 AA, and 164 to 205 AA in both Asian and total samples. D614G, T9I, I82T, and R203M were inferred as the most frequent mutations in S, E, M, and N AASs. Timeline research showed that substitution mutation in the location of 614 among Asian and total S AASs was detected from January 2020.

**Conclusion:**

N protein was the most non-conserved protein, and the most prevalent mutations in S, E, M, and N AASs were D614G, T9I, I82T, and R203M. Screening structural protein mutations is a robust approach for developing drugs, vaccines, and more specific diagnostic tools.

## Introduction

Since the outbreak in December 2019, over 500 million cases and more than 6 million deaths have been reported worldwide as a result of COVID-19 caused by severe acute respiratory syndrome coronavirus 2 (SARS-CoV-2) (1). Airborne aerosol, respiratory droplets, and direct or indirect contact with respiratory droplets are thought to be the primary routes of transmission for SARS-CoV-2 (2). Depending on the geographical area, SARS-CoV-2 mortality can differ significantly. Various factors can contribute to variations in viral infection rates, including national strategies for restricting the movement of people, isolation and quarantine, and genetic differences in population immunity (3). Genetic mutations and evolution capabilities may also affect the viral infection rates as the average number of mutations per sample differs significantly (3, 4).

SARS-CoV-2, a member of the betacoronavirus genus, has a pleomorphic envelope with spikes embedded on its surface (5). About two-thirds of its RNA genome comprises two open reading frames (ORF) called ORF1a and ORF1b, which are cleaved into 16 non-structural proteins (NSPs) necessary for viral replication (5). SARS-CoV-2’s critical structural proteins are spike (S), envelope (E), membrane (M), and nucleocapsid (N), located in the 3’ end of its genome (6). The virus enters human cells via binding its spike glycoprotein, encoded by the S gene, to the human angiotensin-converting enzyme 2 (ACE2) receptor (7). The S protein comprises two subunits called S1 and S2, the former of which is the focal point of major immunogenic epitopes recognized by neutralizing antibodies (8, 9). The E gene encodes the envelope, which is vital for the assembly and release of viruses (10). The M and N genes encode interferon suppressing and nucleocapsid forming proteins, respectively (11, 12). 

RNA viruses evolve fast with a high error rate. As a result, the pathogenicity and transmissibility of SARS-CoV-2 could be altered by mutations in its genome, rendering drug and vaccine development more challenging (13). For example, one of the most common mutations of SARS-CoV-2 is D614G, located in the spike protein, which increases the infectivity of SARS-Cov-2 (14). The high rate of mutations in the S protein has brought about challenges such as decreased neutralization activity against diagnosis and prevention of the disease, as most vaccine platforms target the spike protein (15). In addition to determining drug resistance, immune escape, and pathogenesis-related mechanisms, biological characterization of virus mutations can provide valuable insights (3). This study aimed to obtain further knowledge on structural mutations of SARS-CoV-2 and analyze their evolutionary trends, focusing on Asian countries. Also, the mutational profiles of different regions of Asia were compared. Finally, the results attributed to Asian samples were compared with worldwide samples.

## Materials and Methods

***Sequence extraction from GISAID***

Data was downloaded from GISAID (https://www.gisaid.org/) with permission from Erasmus Medical Center (16-18). The amino acid sequences (AASs) of the SARS-CoV-2 four structural proteins were extracted. All AASs were compared with the reference sequence, the Wuhan-2019 virus (access number: EPI_ISL_402124). The exclusion criteria were as follows: non-human samples, samples that differ in AAs length compared with the reference sequence, and samples with unspecified AAs.

***Mutation tracking using sequence alignment***

SARS-CoV-2 structural protein data extraction, sequence alignment, and mutation detection analysis were performed using Python 3.8.0. The algorithm utilized for detecting mutants is as follows:

For refitem, seqitem in zip (refseq, seq)

If (refitem != seqitem)

***Report a new mutant***

The terms ‘Refseq’ and ‘seq’ in the algorithm refer to the Wuhan-2019 virus and sample sequence, respectively. Mutations with attributed locations and subsets AA were included in the final report.

***Data normalization***

Normalization of the frequencies was applied in order to better compare data in Asia versus worldwide. Therefore, the Asia continent was divided into six regions, including North Asia, West Asia, Central Asia, East Asia, South Asia, and Southeast Asia. As a result, the number of mutations for each Asian country was divided by the number of attributed sequences that were comparable in equal proportions. Microsoft Power BI and R 4.0.3 were used throughout the process.

## Results

***Quantity insight toward mutations***

2083876 samples from Asian countries and 26090908 samples in total were qualified to be imported to the study from the GISAID database. Asian samples involved 106684, 763650, 706808, and 506734 samples for S, E, M, and N AASs, respectively. Moreover, there were 950459 samples for global S AASs, 9914529 global E AASs, 8860463 global E AASs, and 6365457 global samples for N AASs.

**Figure 1:**
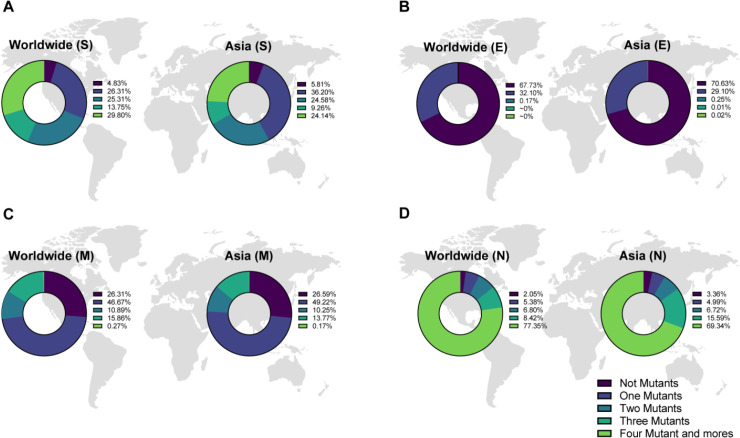
Pie chart plot belonging to the prevalence of mutations among spike (S), envelope (E), membrane (M), and nucleocapsid (N) amino-acid sequences (AASs) in SARS-CoV-2 up to April 2022 in Asia and the World. Sections A, B, C, and D display data attributed to S, E, M, and N AASs, respectively

The results displayed that 5.81% of Asian S samples, 70.63% of Asian E AASs, 26.59% of Asian M samples, and 3.36% of Asian N samples carried no mutations. The rate of carrying one mutation in Asian S samples was determined as 36.20% (Figure 1A). Also, 24.58% of Asian samples attributed to these AASs displayed two mutations, and 9.26% and 24.14% of Asian S proteins showed three, and more than three mutations, respectively. We found that 29.10% of the Asian data belonging to E AASs carried one mutation and 0.25%, 0.01%, and 0.02% of such samples harbored two, three, and more than three mutations, respectively (Figure 1B). The frequency rates of one mutation among Asian M and N proteins were 49.22% and 4.99%, respectively (Figures 1C, 1D). Worldwide data demonstrated that 4.82%, 67.72%, 26.30%, and 2.05% of S, E, M, and N AASs did not display any mutations and 26.31%, 32.10%, 46.67%, and 5.38% of them harbored one mutation, respectively. 

**Figure 2:**
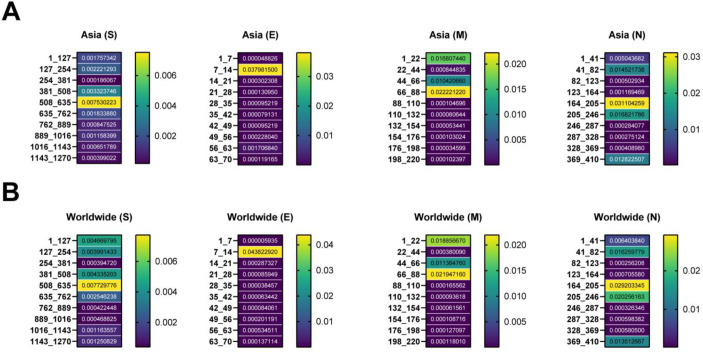
Heat map of mutations among structural proteins of SARS-CoV-2 up to April 2022. The plots show the frequency of mutations per 100 AAs in Asia and the World. The hotspot regions of S, E, M, and S AASs were illustrated in the A, B, C, and D sections, respectively

The regions of 508 to 635 AA (0.0075 frequency), 7 to 14 AA (0.0379 frequency), 66 to 88 AA (0.0222 frequency), and 164 to 205 AA (0.0311 frequency) were introduced as the protein regions with the highest frequent mutations relative to the total AASs among the S, E, M, and N samples in Asia. The mentioned regions were considered the hot spot regions among worldwide S, E, M, and N samples with the frequencies of 0.0077, 0.0438, 0.0219, and 0.0292, respectively (Figure 2). The heat map displayed that the regions of 381 to 508 (0.0033 frequency), 56 to 63 (0.0017 frequency), 1 to 22 (0.0168 frequency), and 205 to 246 (0.0168 frequency) were the second regions among Asian S, E, M, and N AASs, respectively. 

***Substitution mutations and frequencies***

**Figure 3:**
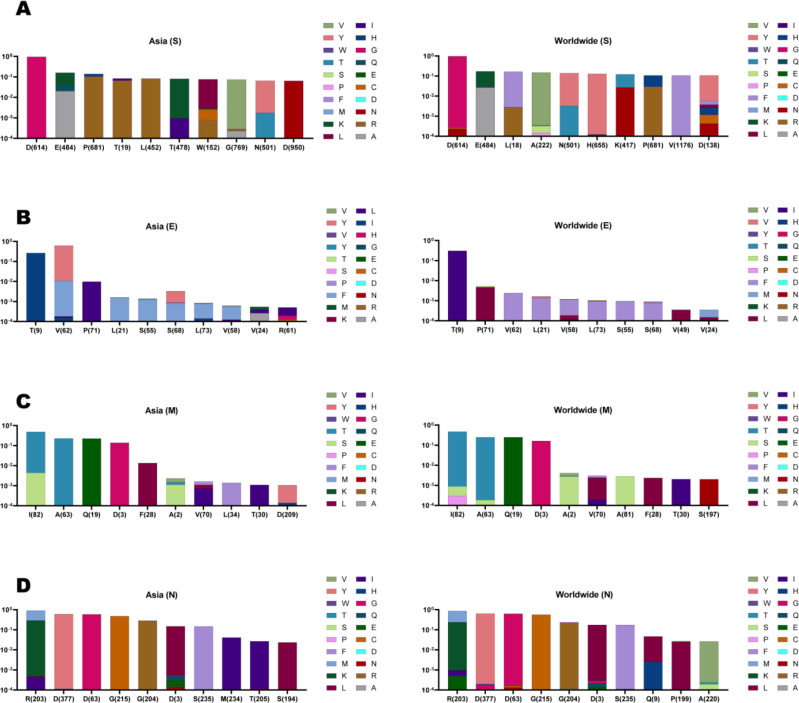
Top ten mutations with the highest frequency among Asian AASs and global samples; the location of altered AAs and substituted AAs are displayed differently based on the frequency rate percentage. For better data representation, the data is represented by a logarithm based on 10

By analyzing the location of mutations, we demonstrated that D614G (0.9530 frequency) was introduced as the first frequent substitution mutation in Asian S AASs, and after that, E484K with 0.1198 frequency rate, P681R with 0.1034 frequency rate, T19R with 0.0667 frequency rate, and L452R with 0.0824 frequency were concluded as the second to fifth prevalent mutations in those S AASs, respectively (Figure 3A). Additionally, T478K (0.0796 frequency), W152L (0.0727 frequency), G769V (0.0732 frequency), N501Y (0.0631 frequency), and D950N (0.0642 frequency) were displayed as the sixth to tenth prevalent structural mutations among Asian S samples, respectively. In Global S AASs, D614G (0.9765 frequency), E484K (0.1407 frequency), L18F (0.1624 frequency), A222V (0.1497 frequency), and N501Y (0.1356 frequency) were the top five prevalent mutations. Regarding the Asian E AASs, T9I (0.2654 frequency), V62F (0.0106 frequency), and P71L (0.0095 frequency) were the top three prevalent mutations, respectively (Figure 3B). Although components of the first three prevalent mutations in E AASs of worldwide data were similar to the Asian sample, their arrangements were different. The global samples of E AASs displayed T9I (with 0.3064 frequency), P71L (with 0.0046 frequency), and V62F (with 0.0022 frequency) as the top three prevalent mutations. Among the Asian samples, all prevalent mutations of E AASs in the positions of fourth to eighth showed AA to phenylalanine (F) substitution. These mutations were as L21F (0.0015 frequency), S55F (0.0012 frequency), S68F (0.0006 frequency), L73F (0.0006 frequency), and V58F (0.0004 frequency), respectively. Moreover, V24A (0.0003 frequency) and R61C (0.0001 frequency) ranked ninth and tenth prevalent mutations, respectively.

On top of that, Asian M AASs displayed I82T (0.4785 frequency), A63T (0.2278 frequency), Q19E (0.2251 frequency), D3G (0.1397 frequency), and F28L (0.0134 frequency) as top five prevalent mutations, respectively (Figure 3C). Subsequently, A2S/V, V70I/F/L, L34F, T30I, and D209Y were the second five most prevalent mutations in Asian M samples with 0.0010/0.0008, 0.0006/0.0005/0.0003, 0.0013, 0.0010, and 0.0009 frequency rates, respectively. Despite different frequencies, global data showed quite similar arrangements of the top four prevalent mutations with the AASs of the Asia continent. The data belonging to Asian N AASs displayed that the R203M/K (0.6149/0.2865 frequencies) mutations ranked first prevalent mutation, and in the following, D377Y (0.6049 frequency), D63G (0.5865 frequency), G215C (0.4770 frequency), and G204R (0.2851 frequency) ranked second to fifth prevalent mutations (Figure 3D). The second five prevalent mutations in Asian N AASs were observed as D3L/Y (0.1459/0.0022 frequencies), S235F (0.1467 frequency), M234I (0.0411 frequency), T205I (0.0267 frequency), and S194L (0.0234 frequency). Supplementary data is available in Frequency.xlsx.

***Mutation frequencies based on the region***

**Figure 4:**
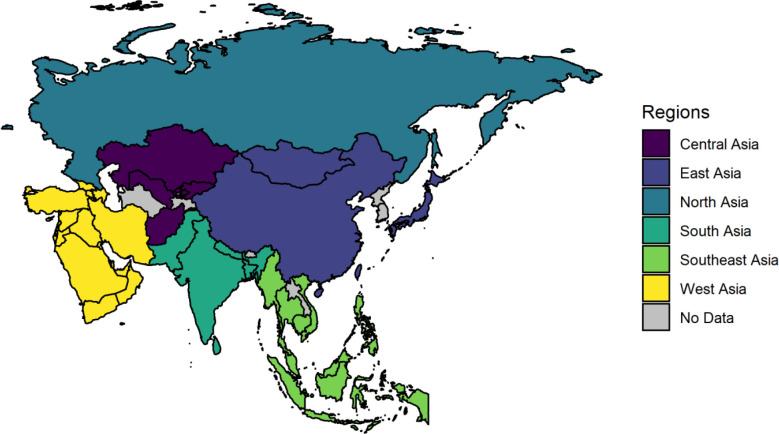
Map of regional divisions in Asia, for better interpretation of the results, Asia was divided into six regions. The countries shown above are those which own submitted AASs with their label

In order to figure out the prevalence of mutations in different parts of Asia, we divided the continent into six regions. The regions and attributed countries are shown in Figure 4. D614G remains the most prevalent S AASs mutation when Asia is divided into six regions; however, three common mutations were not identical in any of these regions. According to Table 1, M153T is the second most prevalent S mutation in North and Central Asia. This mutation did not rank as one of the top ten mutations in other regions, except in East Asia, with the fifth most frequent mutation rank. Despite regional proximity, the mutational profile of S AASs differs significantly between South and Southeast Asia. Except for D614G (0.9688 frequency rate for South Asia and 0.8452 frequency rate for Southeast Asia) and P681R (0.2452 frequency rate for South Asia and 0.3093 frequency rate for Southeast Asia), the third to tenth frequent mutations displayed different arrangements. Besides, N439K, S12F, A701V, and G1251V were the frequent mutations among S AASs of Southeast Asia, which have not been observed in the top ten frequent mutations elsewhere. Other discriminative mutations among S AASs and other structural AASs have been implied in Table 1. On the other hand, E8D, with a frequency rate of 0.0045, was the member of frequent mutations among E AASs observed in North Asia. Mutations of S16G and F23L in West Asia, V70F, N66S, and V75L in Central Asia, V24A in East Asia, V49L, A41V in South Asia, and D72G and S50I in Southeast Asia were other discriminative mutations not seen in other regions. Intriguingly, three of the top E AASs mutations in South and Southeast Asia were identical, and eight of the top ten mutations were the same independent of their order.

Among M AASs, arrangements of the top four prevalent mutations are almost identical among all six regions. Central Asia displayed distinct mutations in this arrangement, including F100I (0.0222 frequency rate) and A2V (0.0035 frequency rate) in the rank of second and fourth frequent mutation, respectively. L16I, S4F, and T208I were frequent mutations in North Asia. Also, there were other discriminative mutations among E AASs; including M1I and A81S attributed to West Asia, F100I, S214I, E167D, and G6V attributed to Central Asia, T30I and M109I attributed to East Asia, and H155Y, I201V, and A40S attributed to Southeast Asia (Table 1). The similarity between frequent mutations among N AASs was demonstrated more than in other structural AASs. The arrangement of first to seventh frequent mutations had a high level of similarity among the regions. G212C (with 0.0335 frequency rate) was the discriminative mutation in the samples of E AASs from North Asia. Q9L in West Asia, A211S and S197L in Central Asia, P151L and Q418H in East Asia, P13L and S413R in South Asia, and L139F in Southeast Asia were other discriminative frequent mutations among E AASs of Asia. Supplementary data about the frequent mutations of these six regions are available in Frequency-regions.xlsx.

***Evolutionary trends based on time***

**Figure 5:**
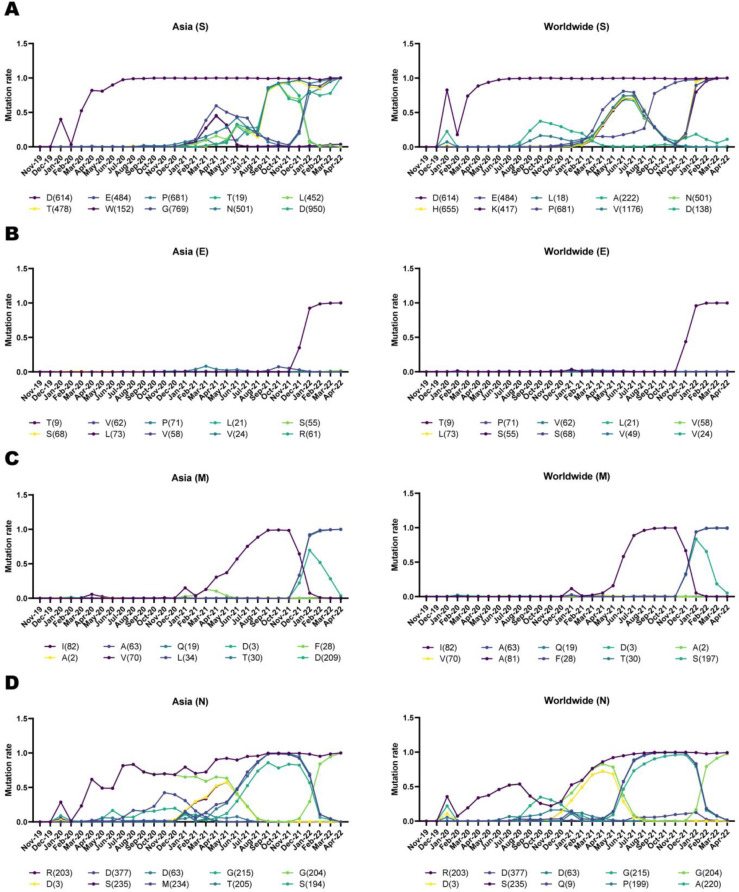
Timeline demonstrates evolutionary trends associated with the top ten frequent mutations of S, E, M, and N of SARS-CoV-2 in Asia and the world. Data is concluded as the numbers of AASs own a mutation over the overall number of AASs, categorized based on the month of sample collection

In order to better study, we identified the patterns of mutation distribution and the evolutionary patterns of their spreading. The timeline distribution pattern of the top ten frequent mutations was displayed in Figure 5. The substitution in the location of 614 among Asian and worldwide S AASs was first detected in January 2020 (Figure 5A). It increased from February 2020 to August 2020 and then sustained maximum frequency until April 2022. Additionally, E484K, P681R, and L452R mutations were detected in March 2020 with facultative evolutionary trends. Evolutionary trends of Asian and worldwide E AASs displayed almost similar distribution patterns for all substitution mutations, except for the T9I mutation. The distribution pattern of T9I mutation increased from November 2021 and reached its maximum frequency after February 2022 (Figure 5B). 

Evolutionary patterns of I82T displayed an almost steady type of distribution up to January 2020. After this time, the frequency of I82T gained and reached its highest frequency in November 2021 (Figure 5C). Like an equilibrium trend, the I82T frequency decreased, and A63T and D3G frequencies increased in November 2021. The arginine mutation (R) in the location of 203 among N AASs had a noticeable growing distribution trend; although it showed oscillating movement from November 2019 to April 2022 (Figure 5D). On top of that, frequency patterns belonging to D377Y, D63G, and G215C mutations displayed almost identical trends. They demonstrated an increasing tendency of distribution from February 2021 and decreasing movement of distribution from November 2021. Additional data is displayed in Timeline.xlsx.

**Table 1:** Top ten frequent substitution mutations in Asia based on the region

**Mutational profile**			**North Asia**	**West Asia**	**Central Asia**	**East Asia**	**South Asia**	**Southeast Asia**
**Frequent mutations**	**S AAS**	1^st^	D614G	D614G	D614G	D614G	D614G	D614G
		2^nd^	M153T	N501Y	M153T	E484K	P681R	P681R
		3^rd^	D138Y^*^	D950N	V16I^*^	W152L^*^	T478K	N439K^*^
		4^th^	Q675H	P681R	V382L^*^	G769V^*^	L452R	S12F^*^
		5^th^	S477N^*^	T19R	T385I	M153T	T19R	A701V^*^
		6^th^	A845S^*^	L452R	Q677H	Q675H	G142D^*^	L452R
		7^th^	A522S^*^	T478K	Q321L^*^	I720V^*^	E484A^*^	T19R
		8^th^	P681H^*^	E484K	N679K	L54F^*^	N440K^*^	T478K
		9^th^	N679K	T95I	E780G^*^	Q677H	T95I	D950N
		10^th^	T385I	K417N^*^	S940F^*^	L5F^*^	D950N	G1251V^*^
	**E AAS**	1^st^	T9I	T9I	T9I	T9I	T9I	T9I
		2^nd^	E8D^*^	P71L	P71L	S55F	V62F	V62F
		3^rd^	P71L	L21F	V58F	V62F	P71L	P71L
		4^th^	S68F	V62F	L21F	L73F	L21F	S55F
		5^th^	V62F	S16G	V70F^*^	V24A^*^	V49L^*^	L21F
		6^th^	L21F	S68F^*^	R69I	P71L	S68F	S68F
		7^th^	L73F	L73F	S68F	S68F	A41V^*^	D72G^*^
		8^th^	R69I	S55F	N66S^*^	V58F	V58F	R61L
		9^th^	S55F	V58F	V62F	L21F	S55F	S50I^*^
		10^th^	T30I	F23L^*^	V75L^*^	R61L	R61L	V58F
	**M AAS**	1^st^	I82T	I82T	I82T	I82T	I82T	I82T
		2^nd^	A63T	A63T	F100I^*^	A63T	A63T	A63T
		3^rd^	Q19E	Q19E	Q19E	Q19E	Q19E	Q19E
		4^th^	D3G	D3G	A2V	D3G	D3G	D3G
		5^th^	L16I^*^	A2V	A63T	F28L	F28L	H155Y^*^
		6^th^	A2V	V70I	S214I^*^	L34F	A2V	I201V^*^
		7^th^	H125Y	A69S	L34F	T30I^*^	V70F	A2V
		8^th^	A69S	M1I^*^	D3G	V70I	A69S	V70I
		9^th^	S4F^*^	D209Y	E167D^*^	M109I^*^	D209Y	L29F
		10^th^	T208I^*^	A81S^*^	G6V^*^	H125Y	L29F	A40S^*^
	**N AAS**	1^st^	R203M	R203M	R203M	R203M	R203M	R203M
		2^nd^	D377Y	D377Y	D63G	D377Y	D377Y	D377Y
		3^rd^	G215C	D63G	D377Y	D63G	D63G	D63G
		4^th^	D63G	G215C	G215C	G215C	G215C	G215C
		5^th^	G204R	G204R	G204R	G204R	G204R	G204R
		6^th^	A211V	D3L	D3L	D3L	S194L	T205I
		7^th^	M234I	S235F	S235F	S235F	P13L^*^	S235F
		8^th^	S235F	T205I	R385K	M234I	R385K	D3L
		9^th^	D3L	Q9L^*^	A211S^*^	P151L^*^	S413R^*^	R385K
		10^th^	G212C^*^	S194L	S197L^*^	Q418H^*^	D3L	L139F^*^

S: Spike; E: Envelope; M: Membrane; N: Nucleocapsid; AASs: Amino-acid sequences

*; This mutation was shown only in this region among the top ten frequent mutations

## Discussion

SARS-CoV-2 was brought to the forefront by the epidemic of pneumonia-like respiratory disease in China and its noticeable high global distribution, which constituted a public health emergency (19). In betacoronaviruses, fluctuations in virulence and infectivity are driven mainly due to genome plasticity via frequent recombination, interspecies transmission, and a high mutation rate. Furthermore, replicating mechanisms prone to errors result in a shift to biological characteristics like the increased transmission capacity (20). Thus, it is essential to study emerging mutations in various geographic regions to comprehend the overall evolutionary trend since the positive selection of any mutation may increase the likelihood of survival rate and jeopardize future diagnostic tests, immunization tools, and therapies for COVID-19 (19).

In the previous study, we explored and conducted the researches with the same approach in order to evaluation mutations frequencies among the Americas. Studying the samples of North America and South America has almost similar parts of result compared to the current research. Similar to the results of Asian samples, the region of 508 to 635 was hotspot region among S AASs from North America; however, hotspot among South America was occurred in the region of 1 to 127 (21). In the present study, analysis of S protein AAs from Asian countries demonstrated that it is the second most non-conserved protein in SARS-CoV-2 after N protein. Like the other world regions, D614G and E484K are the two most prevalent identified mutations in the S glycoprotein (22). Of the ten most frequent mutational spots, eight cases (D614, E48K, P681R, T19R, L452, T478K, W152L, and N501Y) are located in the S1 domain and play role in the pathogenicity of the SARS-CoV-2 (23). None of the top three prevalent S protein mutations were similar in six regions of Asia. But, D614G and P681R substitutions were the same in South and Southeast Asia. Five mutation positions are identical between Asia AAs and other world regions, and three out of ten substitutions of AA residues (E484K, L452, and T478K) have been found in the RBD region. This could pose a significant challenge to existing vaccinations and treatments. The neutralizing activity of BNT162b2 and mRNA-1273 vaccine was evaluated in a recent study against pseudotype viruses containing K417N, E484K, N501Y, and combinations of these three RBD mutations (B.1.351 variant). The neutralizing activity of the K417N mutation variant was not different from the wild type, but it was significantly reduced against the E484K and N501Y mutations and the K417N: E484K: N501Y combination. It highlights the importance of monitoring and surveillance of RBD mutation in the effectiveness of COVID-19 mRNA vaccine and also eliciting long-lasting neutralizing antibodies (9, 22). Likewise, Chen et al. reported that sera from BNT162b2 vaccine recipients showed a decreased capacity to neutralize viruses harboring E484K and N501Y (25). It seems that the E484K alteration was responsible for the neutralization resistance. In addition, it may change the stability, increase binding affinity to host cell receptors, and susceptibility of specific proteins to neutralizing monoclonal antibodies, as well as raise the viral load and transmissibility (26-28). About 60% of the sequences under examination contained simultaneous mutations in the S protein, which may reorganize the protein through the absence of hydrogen bonds with nearby residues and increased interaction of the S1 region with ACE2, enhancing viral infection and transmission. Therefore, these concomitant mutation regions must be precisely investigated. Generally, the distribution of prevalent mutations in Asian samples (508 to 635 AAs) is comparable to that of samples from other regions of the world; however, the mutation types are substantially different (29). The emergence of the D614G variant throughout time suggests that this S mutation has resulted from positive selection during viral evolution and has become fully predominant in Asia and the globe (30). D614G variant appears to have contributed to viral fitness by increasing infectivity, transmissibility, and stability compared with the original strain. Still, it does not appear to have affected disease severity (31).

Furthermore, it has been reported that N501Y and E484K/A mutations contribute to increased binding affinity to the ACE2 receptor and vaccine escape, respectively (32, 33). Also, the beta and kappa SARS-CoV-2 variants share a common mutation, E484K, which accounts for their increased infectiousness and rapid spread (34). Additionally, the timeline analysis shows that the frequency of the four prevalent S protein mutations in Asia (L452, D950, W152, and G769) and the rest of the world (A222, L18, D138, and K417) decreased with time, probably as a result of diminished viral evolution advantages. The RBD mutations including L452R and E484Q mutations were unique to lineages B.1.617.1 (Kappa variant) and B.1.617.3, while L452R and T478K were identified in lineage B.1.617.2 (Delta variant). However, lineage B.1.617.3 was defined by mutations T19R and E484Q (35). 

The current analysis revealed that E proteins are the most conserved structural proteins of SARS-CoV-2, as prior study and more than seventy percent of Asian E AAs do not indicate alterations (36). This proposed that mutations within E genes be minimized, as they may impact viral integrity and life cycle (22). In a previous study, the most common E protein mutation was found to be S68F; however, our research implies that T9I is the most prevalent substitution in six regions of Asia. Additionally, the most frequent mutations that were found were approximately similar throughout South and Southeast Asia. Due to the travel and close contact between the inhabitants of the bordering countries in these two regions of Asia, there may be a transmission strain between the two areas. Due to the hydrophobicity of isoleucine, it is hypothesized that this variant may enhance the interaction with membrane lipids (37). Furthermore, these modifications may impact the performance of real-time RT-PCR-based COVID-19 molecular detection (36, 38). E protein operates by interacting with M and other accessory proteins such as ORF3a and ORF7a, as well as the host cell proteins (39). Of ten prevalent mutations, seven AA mutations were displayed in the C-terminal domain of E protein, which plays a pivotal role in COVID-19 pathogenesis and can alter the E protein’s binding to tight junctions. Even though the E protein is highly conserved, the observed mutations have important biological implications, particularly in therapeutic approaches. Additionally, these mutations can change E-protein’s structural and binding properties (36). The most prevalent observed mutation (T9I) in the E protein was consistent with heat map data (7-14 AAs region), and it also is in accordance with global data (29, 40). But it is partly different from earlier reported common mutations (P71L, S68F, and L73F) (19). Notably, the two most common mutations (L21 and V24) in the transmembrane domain, a key determinant in the pentameric configuration of E protein, were identified in this study (36).

The most prevalent envelope protein, M-Protein, is necessary for viral assembly and morphogenesis. It significantly hinders the immunological response by preventing the formation of type I and type III interferon and blunting the T-cell-driven immune response (41). In order to facilitate coronavirus assembly, it interacts with the envelope and might bind to N protein (42). Eight of the ten prevalent AA modifications were the same in both Asian and global data, and the viral fitness advantage may have contributed to the selection of two changes (A63 and Q19) after November 2021. The most common mutation (I82T) and another widespread mutation (V70) are located in the transmembrane helical domain and are primarily identified in the US, and may be involved in transport function (43). As the prevalence of the I82 mutation decreased between December 2020 and November 2021, the frequencies of the other three mutations (A63, Q19, and D3) climbed; the D3 increase, however, has since leveled out. Furthermore, M mutations have been hypothesized as a probable explanation for the rise in COVID-positive cases, which is more common among younger patients (43, 44). The N protein is the most mutant structural protein in Asia, and approximately seventy percent of AAs displayed at least one mutation. Seven of the ten most prevalent mutations in N AAs from Asia were the same as global mutations. The R203M/K mutation, which has been observed in the Alpha, Delta, and Omicron variants, improves infectivity and confers immunity resistance. It can also speed up the condensation of N protein with the RNA of the virus to promote virion formation (29). In addition, many mutations developed between November 2019 and April 2022, but only two mutations (R203M/K and G204) were ultimately selected. Our research has two drawbacks. In this research, we first investigated AASs without analyzing their nucleotide sequences. This hindered us to examine additional characteristics of newly-emerging variations, such as codon bias. The second constraint was the exclusion of the country-of-origin AAs samples.

## Conclusion

The present study indicates that N and S AASs are the most non-conserved proteins in SARS-CoV-2 in Asia. It was determined that the most prevalent mutations in S, E, M, and N AASs were D614G, T9I, I82T, and R203M and that six regions of Asia shared these substitutions. More genomic surveillance is essential to better understand the developing genetic variants and how they are related to the disease severity. Moreover, it is vital to do extra studies to keep track of emerging new mutations and forestall the development of SARS-CoV-2 strains that are resistant to vaccines and treatment in the future.

## Authors’ Contributions

MM, KR, and MMS Contributed to conceptualization, MA Contributed to study design, KR and MM Designed workflow and code and data analysis. KR and MM Performed data visualization. MA, MK, and RK Wrote the manuscript. MA and MK Monitored the accuracy of additional data. MA and RK Designed graphical content. ZM Edited and supervised the work. All authors reviewed the manuscript.

## Conflicts of Interest

The authors have declared that no conflicts of interest exist.
